# Nigella Sativa reverses osteoporosis in ovariectomized rats

**DOI:** 10.1186/1472-6882-14-22

**Published:** 2014-01-14

**Authors:** Ansam Aly Seif

**Affiliations:** 1 Physiology Department, Faculty of Medicine, Ain Shams University, Abbassiya, Cairo, Egypt; 2 Postal address: 12 Abdullah Abu Elseoud Street, Triumph, Heliopolis, Cairo, Egypt

**Keywords:** Osteoporosis, Nigella sativa, Ovariectomy, Rats

## Abstract

**Background:**

Osteoporosis poses a significant public health issue. It is a skeletal disorder characterized by compromised bone strength that predisposes to increased risk of fracture. There is a direct relationship between the lack of estrogen after menopause and the development of osteoporosis. About 33% of women over 50 will experience bone fractures as a result of osteoporosis. Nigella Sativa (NS) has been shown to have beneficial effects on bone and joint diseases. The present study was conducted to elucidate the protective effect of Nigella Sativa on osteoporosis produced by ovariectomy in rats.

**Methods:**

Female Wistar rats aged 12–14 months were divided into three groups: sham-operated control (SHAM), ovariectomized (OVX), and ovariectomized supplemented with nigella sativa (OVX-NS) orally for 12 weeks; 4 weeks before ovariectomy and 8 weeks after. After 12 weeks, plasma levels of calcium (Ca^+2^), phosphorous (Pi), alkaline phosphatase (ALP), amino terminal collagen type 1 telopeptide, malondialdehyde (MDA), nitrates, nitric oxide surrogate, tumor necrosis factor-α (TNF-α), and interleukin-6 (IL-6) were measured. Histological examination of the liver and the tibia was conducted. Histomorphometric analysis of the tibia was also performed.

**Results:**

OVX rats showed significant decrease in plasma Ca^+2^, accompanied by a significant increase in plasma ALP, amino terminal collagen type 1 telopeptide, MDA, nitrates, TNF-α and IL-6. These changes were reversed by NS supplementation in OVX-NS group to be near SHAM levels. Histological examination of the tibias revealed discontinuous eroded bone trabeculae with widened bone marrow spaces in OVX rats accompanied by a significant decrease in both cortical and trabecular bone thickness compared to Sham rats. These parameters were markedly reversed in OVX-NS rats. Histological examination of the liver showed mononuclear cellular infiltration and congestion of blood vessels at the portal area in OVX rats which were not found in OVX-NS rats.

**Conclusion:**

Nigella sativa reverses osteoporosis in ovariectomized rats, which could be attributed to its high content of unsaturated fatty acids as well as its antioxidant and anti-inflammatory properties.

## Background

The major bone disease is osteoporosis, a systemic skeletal disease characterized by low bone mass and microarchitectural deterioration of bone tissue, with a consequent increase in bone fragility and susceptibility to fracture. The main cause of osteoporosis is menopause or estrogen-deficiency [1]. The increasing evidence of postmenopausal osteoporosis and its related fractures have become global health issues recently [2]. Although hormone replacement therapy has been proven to be efficacious in preventing bone loss, yet, it is not desirable to many women due to its side effects [3].

The use of natural products as an alternative to conventional treatment in healing and treatment of various diseases has been on the rise in the last few decades. Nigella sativa, a natural herb which belongs to *Ranunculaceae* family, has long been used as a natural medicine for treatment of many acute, as well as, chronic conditions. It is also known as black cumin or *habatus sauda.* The seed is the source of the active ingredients of this plant [4]. Nigella sativa seed oils constitute a good alternative source of essential fatty acids compared with common vegetable oils and could contribute to the overall dietary intake. On the other hand, in terms of both quantity and quality, these seeds are potentially attractive source of protein, lipid and some common minerals that appear to have a very positive effect on human health [5]. Nigella sativa seed extracts and its oil have been exploited for their various health benefits. Studies have revealed various therapeutic values of NS such as anticancer, antioxidant, antibacterial, antifungal, antiparasitic and antiasthmatic [6]. Besides that, previous literatures on NS and thymoquinone (TQ), the main active ingredient of NS, have shown that they have beneficial effects on bone and joint diseases [6].

To our knowledge, there is no study of NS or TQ on postmenopausal osteoporosis animal model. In view of the aforementioned data, the present study aimed to test the efficacy of NS in mitigating or prevention of postmenopausal osteoporosis using the ovariectomized rat model.

## Methods

The present study was conducted in the Physiology Department, Faculty of Medicine, Ain Shams University, and approved by Faculty of Medicine Ain Shams University (FMASU), Research Ethics Committee (REC), Cairo, Egypt, which conforms to the Guide for the Care and Use of Laboratory Animals published by the US National Institutes of Health. This study was performed on 30 female Wistar rats aged 12–14 months. Rats were maintained under standard conditions of boarding. They were fed standard laboratory chow with free access to water. Rats were allocated into 3 groups: a) SHAM-operated control (SHAM) rats (n = 10), b) Ovariectomized (OVX) rats (n = 10), c) Nigella Sativa-supplemented Ovariectomized (OVX-NS) rats (n = 10) which received a dose of 800 mg/kg body weight of nigella sativa daily for 12 weeks, 4 weeks before ovariectomy and continued for 8 weeks after the operation. This dose was chosen because it corresponds to the submaximal dose of thymoquinone (TQ), the active ingredient of nigella sativa, producing hypotensive effect in rats [7]. NS seeds (Bioextract (Pvt) Ltd, Sri Lanka, http://www.bioextracts.lk) were provided in the form of 500 mg capsules of grounded NS (powder). The powder was added to distilled water at room temperature to prepare a crude suspension of nigella sativa, a few minutes before giving it to rats by oral gavage. An equivalent volume of water was administered by the same route to the other groups of rats.

Bilateral ovariectomy and sham operation were performed under ether anaesthesia. A midline longitudinal incision was made inferior to the rib cage. The ovaries of rats in groups OVX and OVX-NS were exteriorized, ligated and excised. The SHAM-operated control rats had their dermal integuments, muscles and peritoneum sectioned, without excision of the ovaries.

### Experimental procedures

On the day of the experiments, overnight fasted rats were weighed and injected intraperitoneally with heparin sodium, 1000 IU (B.Braun Melsungen AG.D-34209 Melsungen, Germany). One hour later, rats were anaesthetized with thiopental sodium 40 mg/kg intraperitoneally (Sandoz, GmbH, Kundl-Austria).

#### Biochemical analysis

Blood samples were collected from the abdominal aorta, centrifuged, and then the plasma was subjected to the following assays: calcium (Ca^+2^), phosphorous (Pi), alkaline phosphatase (ALP), amino terminal collagen type 1 telopeptide (NTx), malondialdehyde (MDA), nitrates, tumor necrosis factor-α (TNF-α), and interleukin-6 (IL-6). Tibia and liver specimens were isolated and processed for histological examination.

#### Plasma Ca^+2^, Pi

Plasma Ca^+2^, Pi were measured using standard laboratory methods.

#### Plasma ALP

Plasma ALP was measured by a kinetic photometric test using the Alkaline Phosphatase FS kit supplied by DiaSys Diagnostic Systems GmbH (Germany) according to the method described by Moss and Henderson [8]. Samples were added to supplied regents (Diethanolamine pH 9.8 1.2 mol/L, Magnesium chloride 0.6 mmol/L, p-Nitrophenylphosphate 50 mmol/L), incubated at 25°C for 1, 2 and 3 min and absorbance was read 400–420 nm. Calculations were made from absorbance readings and ALP was expressed as IU/L.

#### Plasma NTx

Plasma NTx was measured by a competitive inhibition enzyme immunoassay (EIA) method using the Osteomark NTx plasma kit supplied by Ostex International, Inc. (USA) according to the method described by Clemens et al. [9]. Plasma controls, test samples, and calibrators were added to the antigen-coated 96-well plate. Antibody to the N-telopeptide cross-links that were conjugated to horseradish peroxidase were then added to each well. The wells were then washed to remove unbound material. Buffered substrate/chromogen reagent was then added to each well. The reaction was stopped by the addition of stopping reagent (1N sulfuric acid), which resulted in a color change from blue to yellow. The absorbance values for the control, calibrators, and test samples were determined spectrophotometrically at 450 nm with a 650 nm reference filter by using a microtiter plate reader. A standard curve was constructed for each assay by plotting absorbance versus concentration for each calibrator. The antigen concentrations of the samples and control were then read from the curve. Assay values were standardized to an equivalent amount of bone collagen and are expressed in nanomoles bone collagen equivalents (nM BCE/L) per liter.

#### Plasma MDA assay

Plasma MDA was determined according to the method of Draper and Hadley [10], based on the reaction of MDA with thiobarbituric acid (TBA). The reaction was performed at 95°C for 15 minutes. The sample was mixed with 2.5 volumes of 10% (w/v) trichloroacetic acid to precipitate the protein. The precipitate was pelleted by centrifugation and an aliquot of the supernatant was allowed to react with an equal volume of 0.67% TBA in a boiling water bath for 15 minutes. After cooling, the absorbance was read at 532 nm.

#### Plasma nitrate assay

Plasma nitrate levels were measured according to the method of Bories and Bories [11], using an enzymatic one step methodology based on the reduction of nitrate by nitrate reductase from Aspergillous species in the presence of β-NADPH and FAD. The concomitant oxidation of the coenzyme β-NADPH was monitored by the decrease in the absorbance at 340 nm. FAD was used as a supplementary electron carrier.

#### Plasma TNF-α assay

Plasma TNF-α was measured by the RayBio® Rat TNF- alpha ELISA kit (RayBiotech, Inc., Norcross, Georgia, USA). Standards and samples were pipetted into the wells and TNF-alpha present in a sample was bound to the wells by the immobilized antibody. The wells were washed and biotinylated anti-Rat TNF-alpha antibody was added. After washing away unbound biotinylated antibody, HRP conjugated streptavidin was pipetted to the wells. The wells were again washed, a TMB substrate solution was added to the wells and color developed in proportion to the amount of TNF-alpha bound. The Stop Solution changes the color from blue to yellow, and the intensity of the color was measured at 450 nm.

#### Plasma IL-6 assay

Plasma IL–6 was measured using using Rat IL-6 ELISA kit (Immuno-Biological Laboratories, Inc.) (IBL-America). Rat IL-6 specific-specific polyclonal antibodies were precoated onto 96-well plates. The rat specific detection polyclonal antibodies were biotinylated. The test samples and biotinylated detection antibodies were added to the wells subsequently and then followed by washing with PBS or TBS buffer. Avidin-Biotin-Peroxidase Complex was added and unbound conjugates were washed away with PBS or TBS buffer. HRP substrate TMB was used to visualize HRP enzymatic reaction. TMB was catalyzed by HRP to produce a blue color product that changed into yellow after adding acidic stop solution. The density of yellow is proportional to the rat IL-6 amount of sample captured in plate.

#### Histological examination of bone

The tibias were dissected out, fixed in 10% buffered neutral formaldehyde, and decalcified in EDTA solution for 2 weeks. Once decalcified, the specimens followed routine histological processing and were embedded in paraffin. Paraffin sections (5 μm thick) from the metaphysis of tibias were deparaffinized and stained by haematoxylin & eosin (H&E) for light microscopic examination [12].

#### Histological examination of the liver

Liver specimens were fixed in 10% buffered neutral paraformaldehyde solution, processed and embedded in paraffin. Thin paraffin sections (5μm) were stained by H&E [12].

#### Morphometric analysis

The mean cortical bone thickness (CBT) and the mean trabecular bone thickness (TBT) were measured in 5 fields/slide from 5 slides for each rat. The reading of each rat was considered as one variable. Measurements were done using the image analyzer (Leica Q 500 MC program) in the Histology Department, Ain Shams University.

### Statistical analysis

Statistical Package for social sciences (SPSS Inc., Chicago, IL, USA) version 16 for windows was used for the statistical evaluation of the results. All data were expressed as mean ± SD. Statistical significance for data was determined using a one-way analysis of variance (ANOVA) with post-hoc test, significance calculated by Tukey test to find inter-group significance. The level of significance was accepted as *P* < 0.05.

## Results

### Biochemical parameters

The results of the present study show that plasma Ca^+2^ levels were significantly decreased in ovariectomized rats compared to both SHAM and OVX-NS rats. NS supplementation in OVX-NS group restored Ca^+2^ levels to be insignificantly different from control SHAM rats, and significantly higher compared to OVX rats. Plasma Pi levels showed no significant changes between the 3 studied groups. Plasma ALP levels were significantly increased in ovariectomized rats compared to SHAM group. Although ALP levels were decreased in OVX-NS compared to OVX rats, these levels remained significantly higher compared to SHAM group. Plasma NTx levels were significantly elevated in OVX rats compared to Sham rats. Plasma NTx levels showed no significant difference between Sham and OVX-NS rats (Table 1).

**Table 1:** **Plasma calcium (Ca**^**+2**^**), phosphorous (Pi), alkaline phosphatase (ALP), malondialdehyde (MDA), nitrates, tumor necrosis factor-α (TNF-α), and interleukin-6 (IL-6) in SHAM-operated control (SHAM) rats, ovariectomized (OVX) rats, and Nigella sativa-supplemented ovariectomized (OVX-NS) rats**

	**SHAM**	**OVX**	**OVX-NS**
Ca^+2^ (mg/dl)	9.20 ± 0.68	7.86 ± 0.45^a,b^	9.09 ± 0.63
	n = 10	n = 10	n = 10
Pi (mg/dl)	3.79 ± 0.24	4.12 ± 0.31	4.00 ± 0.35
	n = 10	n = 10	n = 10
ALP (IU/liter)	58.10 ± 14.56	86.20 ± 9.37^a^	74.30 ± 10.24^a^
	n = 10	n = 10	n = 10
NTx (nM BCE)	10 ± 1.28	28.69 ± 3.02^a,b^	10.84 ± 1.15
	n = 10	n = 10	n = 10
MDA (μmol/l)	1.50 ± 0.19	1.92 ± 0.13^a,b^	1.71 ± 0.24
	n = 10	n = 10	n = 10
Nitrates (μmol/l)	92.70 ± 20.77	142.20 ± 18.56^a,b^	75.50 ± 15.07
	n = 10	n = 10	n = 10
TNF-α (pg/ml)	27.92 ± 2.68	87.62 ± 5.47^a,b^	36.55 ± 4.32^a^
	n = 10	n = 10	n = 10
IL-6 (pg/ml)	18.87 ± 1.26	45.56 ± 4.72^a,b^	29.13 ± 4.60^a^
	n = 10	n = 10	n = 10

n is the number of observations.

^(a)^is the significance calculated by Tukey test, P < 0.05 from SHAM-operated control group.

^(b)^is the significance calculated by Tukey test, P < 0.05 from OVX-NS group.

Plasma MDA levels were significantly increased in OVX rats compared to SHAM rats. These levels were normalized by NS supplementation in OVX-NS group to be insignificantly different from SHAM group and significantly lower than OVX group. Plasma nitrate levels were significantly higher in OVX group compared to both SHAM and OVX-NS groups (Table 1). Plasma TNF-α and IL-6 levels were significantly higher in OVX rats compared to SHAM rats. These levels were significantly decreased by NS supplementation in OVX-NS group compared to OVX rats, though still being significantly higher compared to SHAM rats.

### Histological results

#### Bone

**Figure 1:**
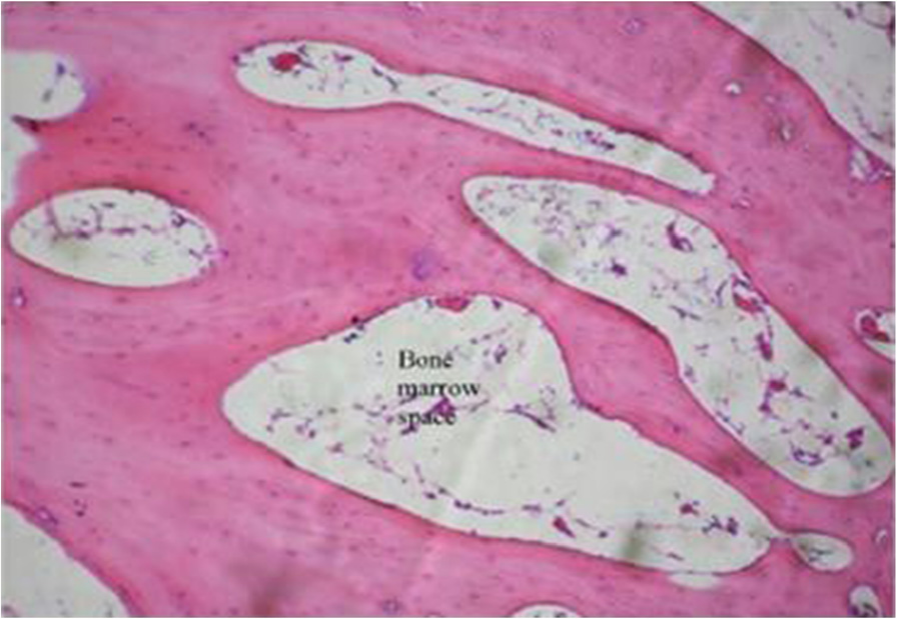
**Proximal metaphysis of tibia of the SHAM group showing network of bone trabeculae of cancellous bone.** Bone marrow fills the spaces between the trabeculae (H&E × 250).

**Figure 2:**
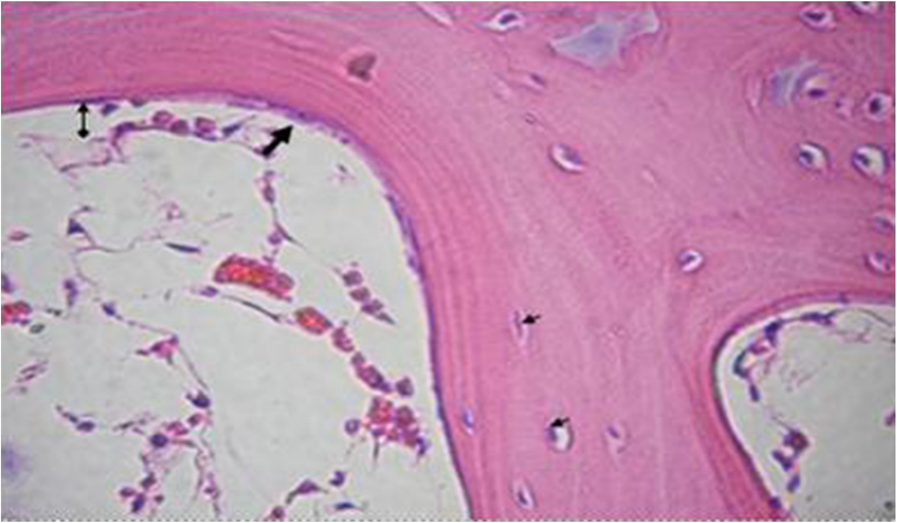
**Proximal metaphysis of tibia of the SHAM group showing bone tabeculae with irrgular bone lamellae and osteocytes residing in their lacunae (a very small arrow), osteoprgenitor cell with flat nucleus (**⬍**) and osteoblast (**⬆**) lining its endosteal surface (H&E × 400).**

**Figure 3:**
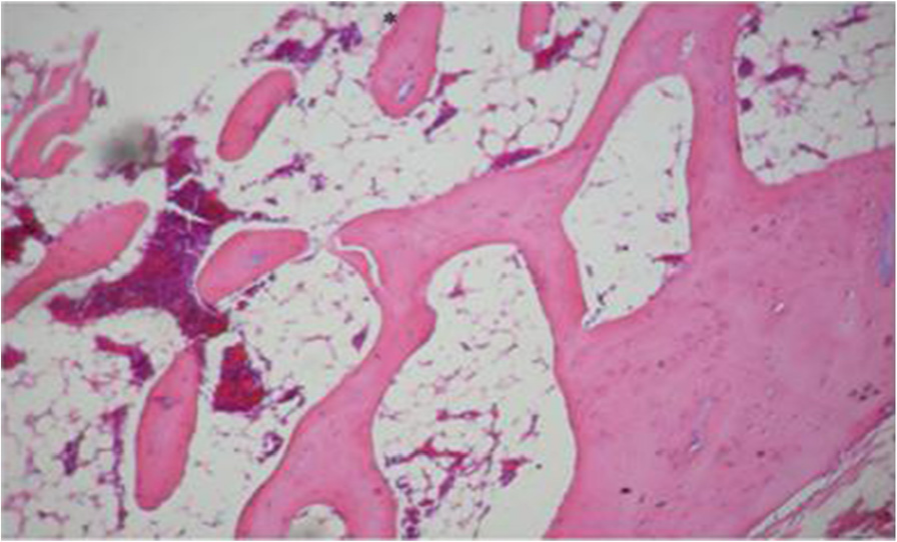
Proximal metaphysis of tibia of the OVX group showing discontinuous network of bone trabaculae with widening of bone marrow spaces (H&E × 200).

**Figure 4:**
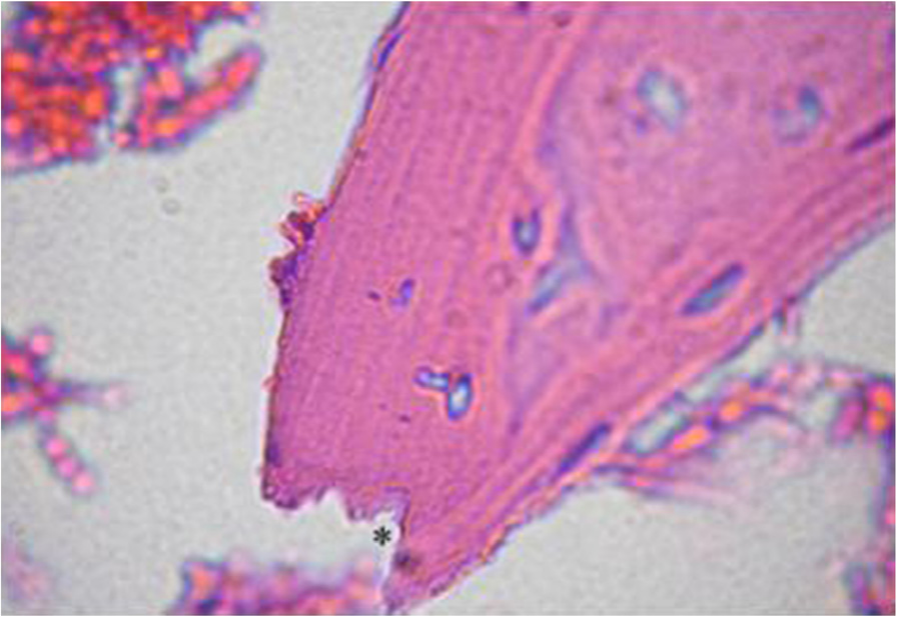
Proximal metaphysis of tibia of the OVX group showing erosion cavities in bone trabeculae (*) (H&E × 1000).

**Figure 5:**
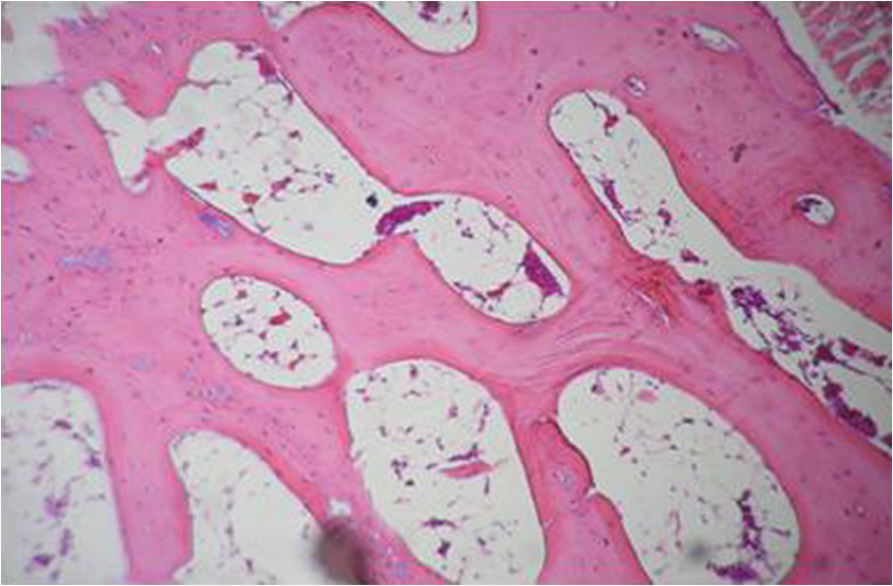
Proximal metaphysis of tibia from OVX-NS group, showing preserved bone architecture as compared to ovariectomized (OVX) rat group (H&E × 200).

The proximal metaphysis of the tibia in SHAM rats showed cancellous bone trabeculae with irregular bone lamellae between which osteocytes resided in their lacunae. The endosteal surface of trabeculae was lined by osteoprogenitor cells with flat nuclei and osteoblasts (Figures 1 & 2). OVX rats showed a discontinuous network of bone trabeculae with eroded cavities and widening of bone marrow spaces (Figures 3 & 4). OVX-NS rats showed marked improvement with preserved bone architecture as compared to OVX rats (Figure 5). The mean CBT and the mean TBT showed a significant decrease in OVX rats when compared to both the Sham and OVX-NS groups. In OVX-NS rats, the mean CBT rats showed no significant change from Sham rats and was significantly higher compared to OVX rats, whereas the mean TBT was significantly higher compared to OVX rats, but still significantly lower compared to the Sham group (Table 2).

**Table 2:** Cortical bone thickness (CBT) and trabecular bone thickness (TBT) in SHAM-operated control (SHAM) rats, ovariectomized (OVX) rats, and Nigella sativa-supplemented ovariectomized (OVX-NS) rats

	**SHAM**	**OVX**	**OVX-NS**
CBT (μm)	238.35 ± 5.41	197.10 ± 6.06^a,b^	236.75 ± 5.84
	(n = 5)	(n = 5)	(n = 5)
TBT (μm)	98.86 ± 2.12	60.78 ± 3.14^a,b^	93.56 ± 2.70^a^
	(n = 5)	(n = 5)	(n = 5)

n is the number of observations.

^(a)^is the significance calculated by Tukey test, P < 0.05 from SHAM-operated control group.

^(b)^is the significance calculated by Tukey test, P < 0.05 from OVX-NS group.

#### Liver

**Figure 6:**
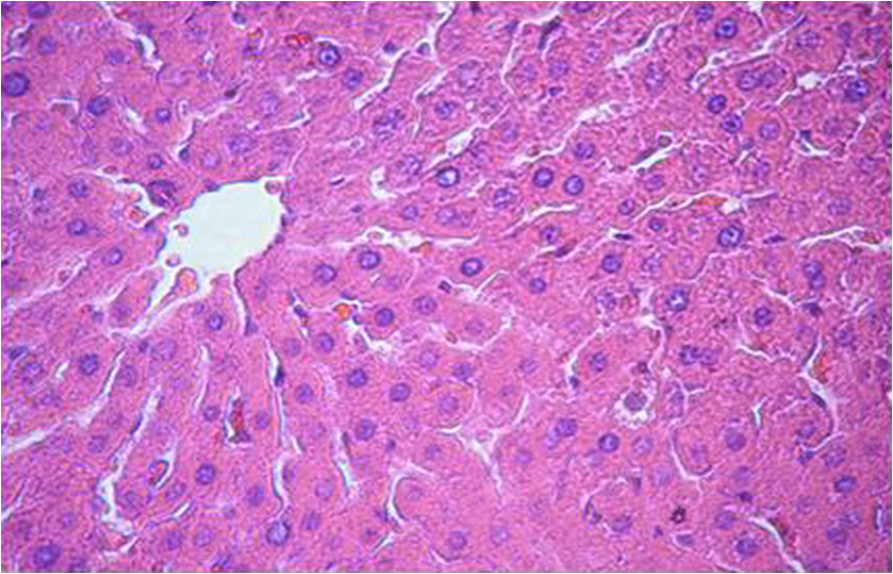
Section in the liver of the SHAM group showing classic parenchymal hepatic lobules with branching and anastomosing cords of hepatocytes radiating from the central vein and separated by blood sinusoids (H&E × 200).

**Figure 7:**
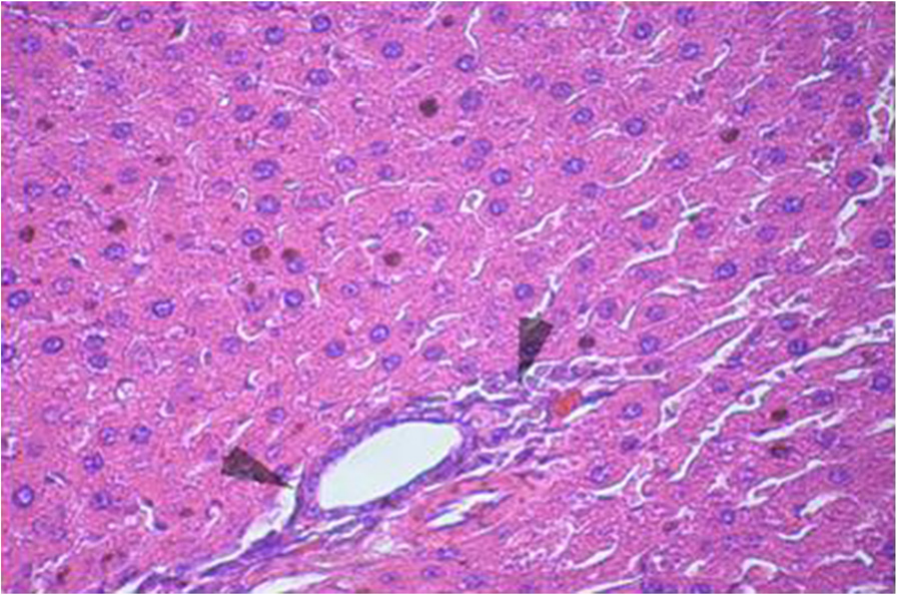
Section in the liver of SHAM group where the portal area shows normal branches of hepatic artery, portal vein and bile duct (H&E × 200).

**Figure 8:**
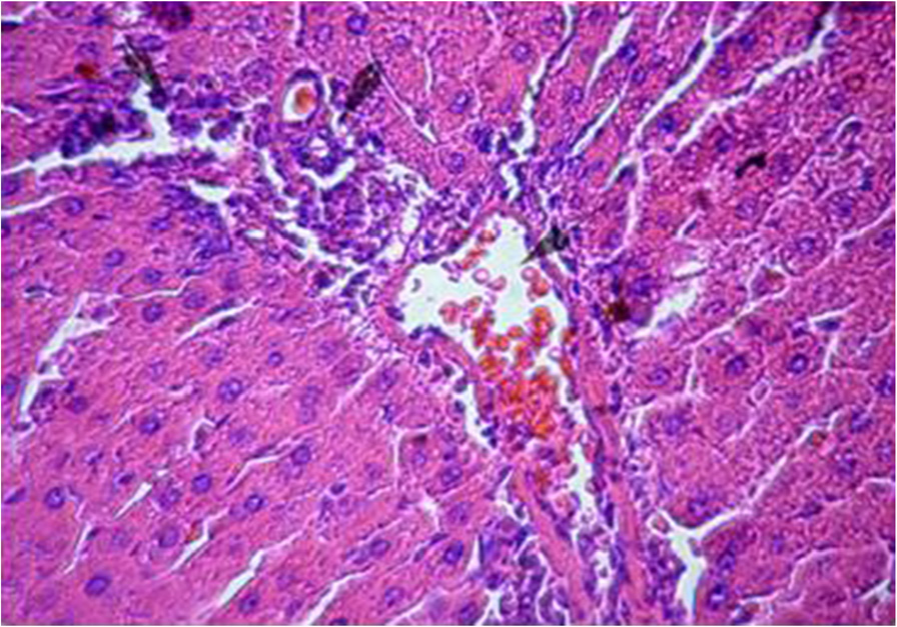
Section in the liver of OVX rats showing mononuclear cellular infiltration and congestion of blood vessels at the portal area (H&E × 200).

**Figure 9:**
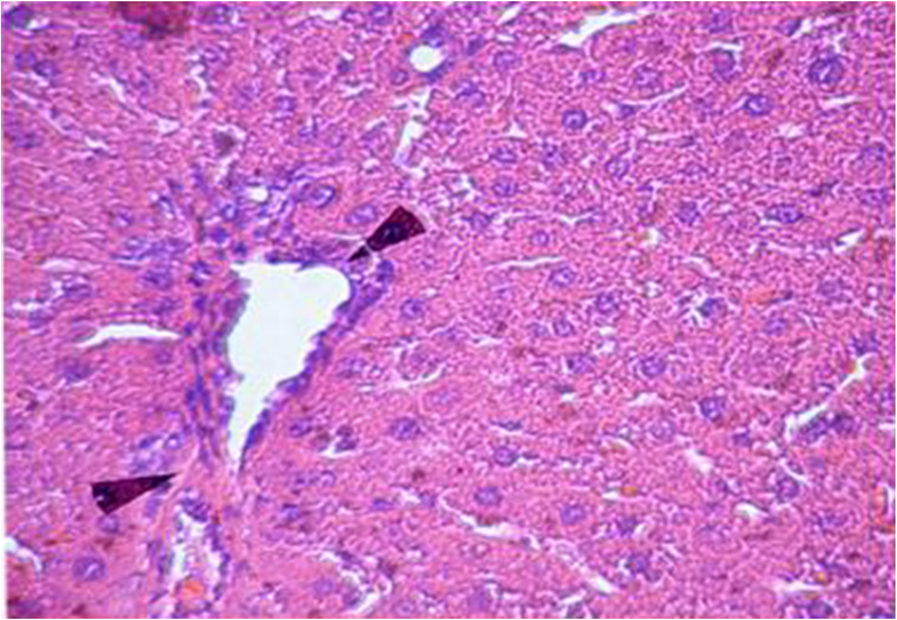
Section in the liver of OVX-NS group showing less congestion of blood vessels at the portal area without mononuclear cellular infiltration (H&E × 200).

Sections from livers of SHAM-operated control rats showed classic parenchymal hepatic lobules with branching and anastomosing cords of hepatocytes radiating from the central vein and separated by blood sinusoids. The portal area shows normal branches of hepatic artery, portal vein and bile duct (Figures 6 & 7). OVX rats showed mononuclear cellular infiltration and congestion of blood vessels at the portal area (Figure 8). In OVX-NS rats, there was less congestion of blood vessels at the portal area without mononuclear cellular infiltration (Figure 9).

## Discussion

The present study highlights the anti-osteoporotic effects of NS and the possible mechanisms behind these effects using ovariectomized rats as a model of post- menopausal osteoporosis. In the present study, ovariectomy in OVX group resulted in significant hypocalcaemia. These results are in agreement with Mattix-Kramer et al. [13], who reported that ovariectomized rats had impaired Ca^+2^ balance that could have contributed to ovariectomy-induced osteoporosis. Moreover, menopause is associated with impaired intestinal Ca^+2^ absorption that could be attributed to reduced plasma 1,25 dihydroxyvitamin D levels, as well as to the resistance of the gastrointestinal system to the action of 1,25 dihydroxyvitamin D [14]. Estrogen has been shown to modulate the end organ effect of 1,25 dihydroxyvitamin D on intestinal calcium absorption [15]. Furthermore, menopause is associated with increased renal excretion of calcium [16]. Estradiol increases renal tubular Ca^+2^ reabsorption [17]. Our results show that NS supplementation in OVX-NS was effective in preventing hypocalcemia caused by ovariectomy in OVX rats. Ali et al. [5] reported that NS seed oils revealed higher degree of unsaturation and that the major unsaturated fatty acids were linoleic acid followed by oleic acid. Oleic acid was found to increase Ca^+2^ levels [18]. Oleic acid was also found to help maintain bone health and prevent calcium loss by promoting the absorption of nutrients in the body [19]. Martin-Bautista et al. [20], reported improvement of bone formation biomarkers after 1-year consumption with milk fortified with oleic acid and other fortifiers with a significant increase in plasma calcium (4%), vitamin D (11%), and osteocalcin (22%). Current evidence in animals also suggests that linoleic acid may help decrease bone loss by enhancing calcium absorption [21]. An average woman is estimated to eat about 2 1/2 lbs of food a day, therefore 0.1–1% of her diet would amount to about 1.14–11.4 g of linoleic acid a day. Given that dietary linoleic acid intakes in men and women are reported to not exceed 500 mg a day [22], theoretically the health benefits attributed to linoleic acid would not likely be observed by diet alone, but would require supplementation. Moreover, Ali et al., [5], reported that NS seeds contain useful quantities of calcium which makes them a natural source of calcium supplementation for pregnant and lactating women as well as for children and elderly people, which might partially explain raised Ca^+2^ levels seen in OVX-NS rats.

In the current study, plasma ALP was significantly increased in OVX rats compared to SHAM rats, indicating increased osteoblastic activity with increased bone formation. OVX rats also showed a significant increase in plasma NTx levels compared to Sham rats indicating increased bone resorption. Moreover, tibias of OVX rats showed eroded cavities with widened bone marrow spaces indicating increased bone resorption. The mean CBT and TBT were both significantly decreased in OVX rats compared to Sham rats. These results are in accordance with Grassi et al. [23], who linked estrogen deficiency to accelerated bone remodeling, where bone resorption outpaced bone formation. NS supplementation in OVX-NS rats lowered plasma ALP levels, albeit still significantly higher compared to SHAM rats, indicating more stable bone formation [6]. NS also lowered plasma NTx levels to levels of control Sham rats, indicating decreased bone resorption to normal control levels. The enhanced effects of NS supplementation are assured by the elevation of the mean CBT to control levels, as well as the elevation of the mean TBT to be very near to control Sham rats, although still significantly lower.

Besides that, previous literatures on NS and TQ have highlighted two properties that might be responsible for their antiosteoporotic effects, that is, antoxidative and antiinflammatory properties [6]. Histological examination of liver specimens showed mononuclear cellular infiltration and congested blood vessels in OVX-NS rats. Recently, plenty of evidences had surfaced, linking inflammation to osteoporosis. This has led to the opinions that inflammation may contribute to osteoporosis [24]. Kireev et al. [25], reported that pro-inflammatory cytokines TNF-alpha *(TNF- α)*, IL-1beta and IL-6 measured in liver homogenates were significantly increased and anti-inflammatory IL-10 decreased during ageing and after ovariectomy in rats. Moreover, Levels of lipid peroxides in the liver homogenates as well as iNOS protein expression and NO levels were increased in old rats as compared to young animals; this effect was more evident in ovariectomized animals. The authors also stated that administration of the different hormonal replacement therapies was able to inhibit the induction of pro-inflammatory cytokines and iNOS, decreased the levels of oxidative stress markers and had therapeutic potential in the prevention of liver injury. Moreover, Pighon et al. [26], showed that exercise training in ovariectomized rats acted like estrogen in properly regulating the expression of inflammatory biomarkers in liver of OVX rats. TQ was believed to exert anti-inflammatory effects by inhibiting the synthesis of prostaglandins and leukotrienes which are the main mediators of inflammation [27]. The results of the present study show significant elevation of plasma nitrates, NO surrogate, in OVX rats compared to SHAM rats, which was lowered to control levels by NS supplementation in OVX-NS group. NO might have been produced by macrophages as one of the inflammatory signs that were corrected by NS supplementation. These results are in agreement with Suna et al. [28], who stated that a possible anti-inflammatory mechanism of TQ might be suppression of nitric oxide production by macrophages. Another study has shown that the alveolar bone loss due to periodontitis was reduced by gastric feeding of TQ to rats. This was accompanied by reduction in osteoclast number and raised osteoblastic activity in TQ-treated rats [29]. In studies using rheumatoid arthritis model, TQ was reported to reduce the serum levels of IL-1 and *TNF-α,*[30]. The production of cytokines including TNF and IL-17 can increase osteoclastogenesis and bone loss in inflammatory liver conditions [31]. Bone protective effects of estrogen might involve suppression of inflammatory cytokines such as IL-1 and *TNF-α,* which in turn activate inducible NO synthase (iNOS). iNOS is only expressed in response to inflammatory stimuli, and NO derived from this pathway potentiates cytokine and inflammatory-induced bone loss [32]. This might be a possible explanation for the detected significant increase in plasma nitrates level in OVX rats in the present study. Furthermore, a previous study reported that IL-1 and *TNF-α* were increased in healthy premenopausal women who underwent ovariectomy and reached the highest levels 8 weeks after ovariectomy, and these changes were associated with indices of bone resorption [3]. In the present study, the anti-inflammatory effects of NS were further assured by the significant decrease in plasma TNF-α and plasma IL-6 levels in the OVX-NS group compared to the OVX group.

Osteoporotic patients were found to be under oxidative stress as their lipid peroxidation levels were elevated and antioxidant enzymes reduced [33,34]. Most risk factors for osteoporosis were associated with oxidative stress such as hypertension [35], diabetes mellitus [36], and smoking [37]. Reactive oxygen species could also stimulate osteoclast formation and activity [38], impair osteoblastic function [39], and decrease osteoblast recruitment and collagen synthesis [40]. Exposure to oxidative stress would result in reduction of bone-mineral density [41]. Increased oxidative stress could be attributed to the loss of the antioxidant effects of estrogen [3]. Since it is apparent that oxidative stress may lead to osteoporosis, antioxidants may play a role in protecting bone against the damaging effects of free-radicals. It has been reported that the free radical scavenging capability of TQ is as effective as superoxide dismutase [41]. It is most effective in scavenging superoxides, the reactive oxygen species which plays an important role in the activation of osteoclasts [42]. The results of the present study are in agreement with the previous data showing that plasma MDA levels, an important measure of lipid peroxidation, were significantly increased in OVX rats compared to both SHAM and OVX-NS rats.

## Conclusion

It can be concluded that NS has shown potential as a safe and effective antiosteoporotic agent, which can be attributed to its high content of unsaturated fatty acids as well as its antioxidant and anti-inflammatory properties.

## Competing interests

I declare that I have no competing interests.

## Pre-publication history

The pre-publication history for this paper can be accessed here:

http://www.biomedcentral.com/1472-6882/14/22/prepub
